# Supplementary material for: Treatment Sequencing and Outcome of Chronic Lymphocytic Leukemia Patients Treated at Fondazione Policlinico Universitario Agostino Gemelli IRCCS: A Thirty-Year Single-Center Experience
Source: Cancers (Basel). 2023 Nov 26;15(23):5592. doi: 10.3390/cancers15235592 (PMC10705134; doi:10.3390/cancers15235592)
Supplement: Supplementary file 1 [file cancers-15-05592-s001.zip › cancers-2683285-supplementary.pdf]

Supplementary Materials

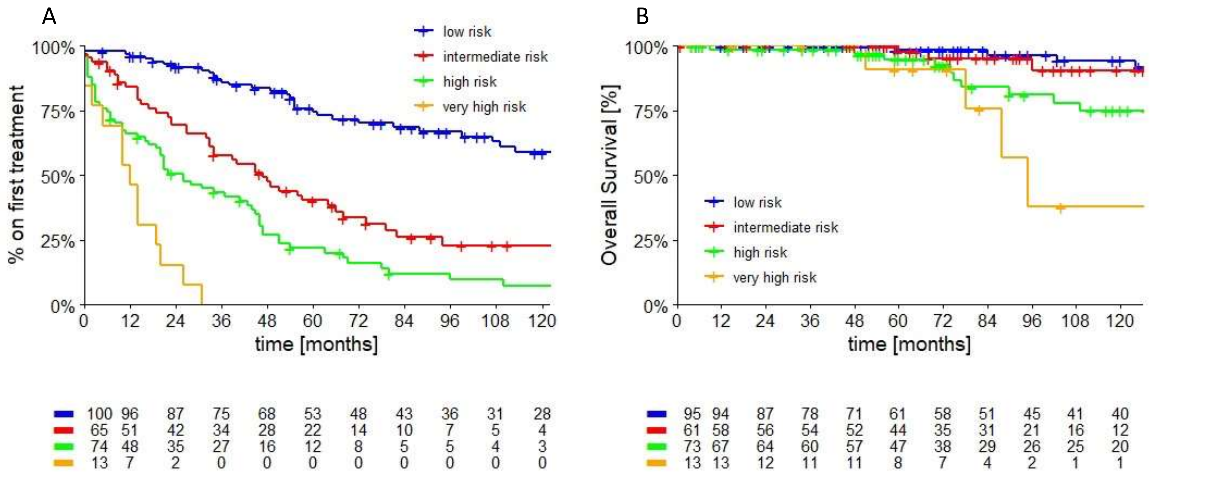

Supplementary Figure S1. Patients' TTFT (A) and OS (B) according to CLL-IPI at diagnosis.

Line of therapy	Treatment	Haematological toxicity	p	Extra-haematological toxicity	p
First line	***cBTKi +/- antiCD20***	6 (8.0)	0.13	34 (45.3)	0.86
	BCL2i +/- antiCD20	3 (21.4)		6 (42.9)	
	CT+antiCD20	57 (35.2)		53 (32.7)	
	CT	6 (11.3)		5 (9.4)	
	other	0		2 (16.7)	
Second line	***cBTKi +/- antiCD20***	10 (18.2)	0.002	24 (43.6)	0.18
	BCL2i +/- antiCD20	9 (56.3)		4 (25.0)	
	CT+antiCD20	9 (24.3)		6 (16.2)	
	CT	1 (7.7)		4 (30.8)	
	other	2 (18.2)		6 (54.5)	
Third line	***cBTKi +/- antiCD20***	6 (26.1)	0.45	9 (39.1)	0.17
	BCL2i +/- antiCD20	6 (37.5)		3 (18.8)	
	CT+antiCD20	1 (16.7)		3 (50.0)	
	CT	2 (40.0)		1 (20.0)	
	other	1 (7.1)		13 (92.9)	

Beyond third line	*cBTKi +/- antiCD20*	0	NA	0	NA
	BCL2i +/- antiCD20	5 (41.7)		4 (33.3)	
	CT+antiCD20	0		0	
	CT	0		0	
	other	1 (20.0)		1 (20.0)	

Supplementary Table S1. Toxicities in the different treatment cohorts

Cohort	Line of therapy	Number of patients	Need for subsequent treatment or death	Deaths	Retreatments
**cBTKi-exposed**	First line	77/157 (49.0%)	8 (10.4%)	16 (10.2%)	4 (2.5%)
	Second line	55/157 (35.0%)	23 (41.8%)		
	After second line	28/157 (17.8%)	19 (67.9%)		
**BCL2i-exposed cBTKi-naïve**	First line	13/34 (38.2%)	1 (7.7%)	7 (11.7%)	4 (11.8%)
	Second line	12/34 (35.3%)	3 (25.0%)		
	After second line	11/34 (32.3%)	3 (27.3%)		
**Double-exposed**	BCL2i after cBTKi	25/26 (96.1%)	10 (38.5%)	4 (15.4%)	/
	cBTKi after BCL2i	1/26 (3.9%)			

Supplementary Table S2. Characteristics of the patients treated with targeted agents. Only adverse events of cBTKi and BCL2i were compared.
